# Modulation Role of Abscisic Acid (ABA) on Growth, Water Relations and Glycinebetaine Metabolism in Two Maize (*Zea mays* L.) Cultivars under Drought Stress

**DOI:** 10.3390/ijms13033189

**Published:** 2012-03-08

**Authors:** Lixin Zhang, Mei Gao, Jingjiang Hu, Xifeng Zhang, Kai Wang, Muhammad Ashraf

**Affiliations:** 1 College of Life Sciences, Northwest A & F University, Yangling 712100, Shaanxi, China; E-Mails: zhanglixinyangling88@yahoo.com.cn (L.Z.); gaomei24@yahoo.com.cn (M.G.); hujingjiang@yahoo.cn (J.H.); zhangxifeng05@yahoo.com.cn (X.Z.); wangkai20@yahoo.cn (K.W.); 2 State Key Laboratory of Soil Erosion and Dryland Farming, Institute of Water and Soil Conservation, Chinese Academy of Sciences, Yangling 712100, Shaanxi, China; 3 Department of Botany, Faculty of Sciences, University of Agriculture, Faisalabad 38040, Pakistan

**Keywords:** abscisic acid (ABA), drought stress, plant growth, glycinebetaine metabolism, maize

## Abstract

The role of plant hormone abscisic acid (ABA) in plants under drought stress (DS) is crucial in modulating physiological responses that eventually lead to adaptation to an unfavorable environment; however, the role of this hormone in modulation of glycinebetaine (GB) metabolism in maize particularly at the seedling stage is still poorly understood. Some hydroponic experiments were conducted to investigate the modulation role of ABA on plant growth, water relations and GB metabolism in the leaves of two maize cultivars, Zhengdan 958 (ZD958; drought tolerant), and Jundan 20 (JD20; drought sensitive), subjected to integrated root-zone drought stress (IR-DS) simulated by the addition of polyethylene glycol (PEG, 12% w/v, MW 6000). The IR-DS substantially resulted in increased betaine aldehyde dehydrogenase (BADH) activity and choline content which act as the key enzyme and initial substrate, respectively, in GB biosynthesis. Drought stress also induced accumulation of GB, whereas it caused reduction in leaf relative water content (RWC) and dry matter (DM) in both cultivars. The contents of ABA and GB increased in drought-stressed maize seedlings, but ABA accumulated prior to GB accumulation under the drought treatment. These responses were more predominant in ZD958 than those in JD20. Addition of exogenous ABA and fluridone (Flu) (ABA synthesis inhibitor) applied separately increased and decreased BADH activity, respectively. Abscisic acid application enhanced GB accumulation, leaf RWC and shoot DM production in both cultivars. However, of both maize cultivars, the drought sensitive maize cultivar (JD20) performed relatively better than the other maize cultivar ZD958 under both ABA and Flu application in view of all parameters appraised. It is, therefore, concluded that increase in both BADH activity and choline content possibly resulted in enhancement of GB accumulation under DS. The endogenous ABA was probably involved in the regulation of GB metabolism by regulating BADH activity, and resulting in modulation of water relations and plant growth under drought, especially in the drought sensitive maize cultivar JD20.

## 1. Introduction

Plants are frequently exposed to a variety of abiotic stresses such as drought stress (DS), which hamper plant growth and crop productivity worldwide [1]. Drought stress (DS) causes considerable yield reduction in most crops including maize. Maize *(Zea mays* L.) is an important cereal crop in northern China which is sensitive to drought [2,3]. Understanding how plants tolerate these stresses is a prerequisite for developing strategies to improve plant stress tolerance [4].

Plants sense and adapt to different stresses by altering their physiological metabolism, and growth pattern, and mobilizing various defense mechanisms [5]. Therefore, accumulation of osmolytes is a prerequisite for osmotic adjustment of all organisms under DS [6]. It is well established that glycinebetaine (GB) accumulates in plants during their adaptation to various types of environmental stresses including drought [7,8]. Glycinebetaine, a quaternary ammonium compound, is a very effective compatible solute which is found in a wide range of crops [7]. In maize, one of GB accumulators, this compatible solute accumulates in leaves in response to water deficit [7,9]. Glycinebetaine has been reported to synthesize from its precursor choline by a two-step oxidation, via the intermediate betaine aldehyde. The first oxidation step is catalyzed by choline monooxygenase (CMO, EC 1.14.15.7), and the further oxidation to GB is catalyzed by betaine aldehyde dehydrogenase (BADH, EC 1.2.1.81), the enzymes involved in GB biosynthesis [10,11].

Abscisic acid (ABA) plays an important role in physiological adaptation of plants to drought stress [12–14]. It has been reported that ABA is not directly involved in modulation of cell enlargement and division [15–17], but it indirectly regulates plant growth by improving stomatal resistance to control transpiration and CO_2_ uptake [13,15,16,18]. These ABA-induced adaptive changes can be of great importance for the survival and better growth of plants under unfavorable environmental conditions [17,19,20]. Although varied roles of ABA are well documented [21,22], it remains unclear how this hormone coordinately regulates GB metabolism in relation to BADH activity and choline content, and in turn plant growth of different maize cultivars using both exogenous ABA and fluridone (Flu), a direct inhibitor of ABA synthesis [23,24].

Keeping in view the above facts, we hypothesized that plant hormone ABA can compensate for drought-induced retardation in the growth of two maize cultivars *i.e.*, Zhengdan 958 and Jundan 20 by employing up-regulation of constitutive GB metabolism to mediate plant adaptation to DS. Previously, the former cultivar showed relatively higher drought index as well as dry matter production and grain yield under drought. Thus, Zhengdan 958 was ranked as drought tolerant and Jundan 20 as drought sensitive [25–26]. With this aim, we designed hydroponic experiments to clarify the responses of maize to exogenous ABA and the ABA synthesis inhibitor Flu with respect to growth and GB metabolism in maize plants subjected to integrated root-zone DS.

## 2. Results and Discussion

### 2.1. Modulation Role of Abscisic Acid (ABA) on the Growth and Leaf Water Relations of Maize Seedlings under Drought Stress

Twelve days of integrated root-zone DS (IR-DS) induced by PEG significantly caused a decline in the growth of seedlings of both maize cultivars. The shoot dry weights (SDW) of Zhengdan 958 (ZD958) and Jundan 20 (JD20) under IR-DS were only 74% and 61% of those of the same two cultivars, respectively, under the controls in the absence of both exogenous ABA and fluridone (Flu), 89% and 73%, respectively, in the presence of ABA, and 67% and 65%, respectively, in the presence of Flu. The leaf relative content (RWC) of ZD958 and JD20 treated with PEG alone had 90% and 87% of the controls, 96% and 91% with both PEG and Flu, and 87% and 82% with both PEG and ABA. Regardless of ABA or Flu treatment, ZD958 showed higher values of SDW and leaf RWC than those of JD20 under IR-DS (Table 1).

Exogenous ABA increased but Flu decreased the SDW of ZD958 and JD20 by 16% and 20%, and 17% and 14%, respectively under IR-DS treatments. As for leaf RWC, application of ABA caused increase while Flu induced decrease in ZD958 and JD20 by 4.8% and 5.1%, and 4.4% and 3.8%, respectively. However, under the controls, ABA or Flu application had no obvious impact on SDW and leaf RWC (Table 1).

### 2.2. The Accumulation Pattern of Endogenous ABA and Glycinebetaine (GB) in Maize Seedlings under Drought Stress

**Figure 1:**
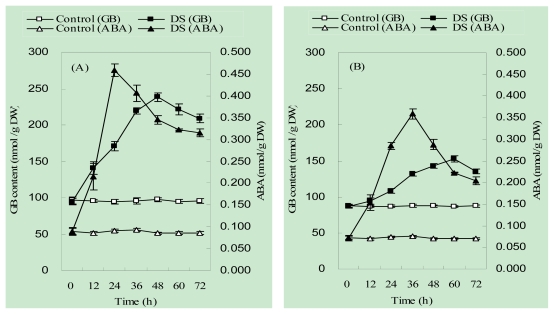
Effects of drought treatment on contents of endogenous ABA and glycinebetaine (GB) in the seedlings of Zhengdan 858 (**A**) and Jundan 20 (**B**) cultivars.

Endogenous ABA and GB in both cultivars accumulated with prolonged period of IR-DS treatment. These responses were more predominant in ZD958 than that in JD20. The ABA contents in ZD958 and JD20 reached their maximum after 24 and 36 h of the start of DS treatment, respectively, being 540% and 480% of those of the control plants. In contrast, GB contents in ZD958 and JD20 were maximum after 48 and 60 h of DS treatment, being 250% and 180% of those of the control plants, respectively. The maximum accumulation of ABA took place earlier than that of GB in the leaves of both drought-stressed maize cultivars (Figure 1).

### 2.3. Modulation Role of ABA on Endogenous ABA and Glycinebetaine (GB) Accumulation in the Leaves of Maize Plants under Drought Stress

**Figure 2:**
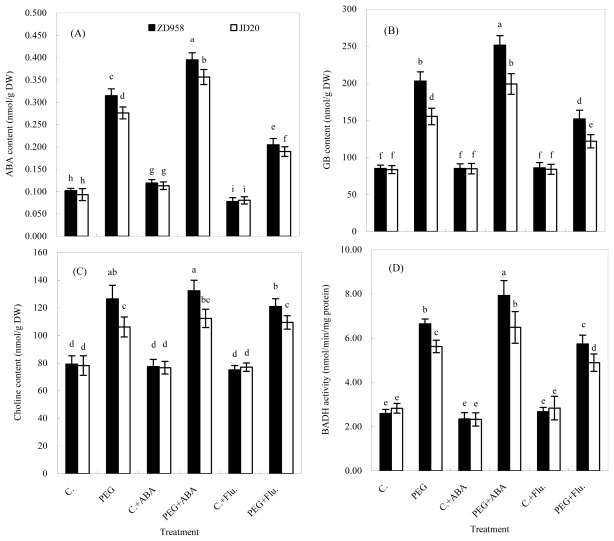
Effects of exogenous ABA and fluridone (Flu) on endogenous ABA content (**A**), glycinebetaine (GB) content (**B**), choline content (**C**) and betaine aldehyde dehydrogenase (BADH) activity (**D**) in the seedlings of two maize cultivars under drought stress induced by PEG and control (**C**). The same letters on the top of the column within each variable are not significantly different among twelve treatments at the 0.05 level.

The accumulation of endogenous ABA and GB was affected by the exogenous application of ABA or Flu in maize plants subjected to IR-DS. In the absence of ABA or Flu, endogenous ABA and GB levels increased 210% and 140% in ZD958 and 190% and 90% in JD20, respectively under IR-DS. The corresponding values in the presence of ABA and Flu were 230% and 160% in ZD958 and 210% and 130% in JD20 for ABA level as well as 200% and 70% in ZD958 and 140% and 50% in JD20 for GB level, respectively (Figure 2A,B).

Addition of ABA and Flu caused increases and decreases, respectively, in endogenous ABA level of drought-stressed plants by 26% and 34% in ZD958, and 29% and 31% in JD20 as well as in GB level by 23% and 25% in ZD958, and 28% and 21% in JD20, respectively, as compared with those in plants without ABA or Flu application. As for the controlled plants, application of ABA and Flu also increased and decreased the endogenous ABA level of both cultivars. However, the increase/decrease rates became less than those in drought-stresses plants. Additionally, ABA and Flu treatments had no significant impacts on GB level in the controlled plants (Figure 2A,B).

### 2.4. Modulation Role of ABA on GB Metabolism in the Leaves of Maize Plants under Drought Stress

Glycinebetaine metabolism determination can be done by assessing the amount of its precursor choline and the activity of the key enzyme BADH. The choline content and BADH activity in the leaves are shown in Figure 2C,D. The choline content and BADH activity increased by 59% and 156% in ZD958, and 37% and 99% in JD20 without ABA or Flu application, by 70% and 237% in ZD958, and 48% and 178% in JD20 with ABA application, and by 61% and 115% in ZD958, and 40% and 72% in JD20 with Flu application respectively, due to drought treatment.

Exogenous ABA increased but Flu decreased BADH activity in both cultivars under IR-DS. The beneficial effects of ABA/negative effects of Flu on BADH activity were greater/less in JD20 than those in ZD958. However, these responses were not recorded under control conditions. As for choline content, there were no significant impacts of added ABA/Flu under both IR-DS and control treatments. However, choline content in plants treated with ABA was greater than that in plants treated with Flu under IR-DS.

### 2.5. Correlations among All Parameters Measured

Correlation coefficients among all traits evaluated were significantly higher under DS than those under control. Importantly, correlations among DM, RWC and content of ABA and choline as well as BADH activity were evident under DS but not for those under control treatment (Table 2).

### 2.6. Interaction of Exogenous ABA or Flu Treatment and Water Regimes as well as Correlation Coefficients for All Parameters Measured

Water regimes and exogenous ABA or Flu treatments had significant effects on all parameters (Table 3). The magnitudes of *F* values across the above parameters were in the order: water regime > exogenous ABA (Flu) > cultivars except choline content. The interaction effects among the above treatments were also mostly significant for all response variables except Cv × A and W × Cv × A as well as W × Cv × Flu for choline content and BADH activity.

### 2.7. General Discussion

Drought stress (DS) is one of the most important abiotic stresses which severely affect crop growth and productivity [1]. Relative water content (RWC) in plant leaves is contemplated as a potential indicator of plant water status, because it is involved in the metabolic activity in tissues. Decline in RWC reflects a loss of turgor that results in limited cell expansion and thereby reduced growth in crop plants [3,4]. Drought stress has been widely reported to cause perturbance in water homeostasis [4]. Decline in the availability of water in plant body leads to molecular damage, growth inhibition and even death [4,5]. It has already been reported that different crop cultivars show varying response to DS for different duration in view of water status and plant growth [8]. The present studies have shown that integrated root-zone DS (IR-DS) caused a decrease in leaf RWC and shoot biomass in maize plants. However, improved RWC and shoot dry matter (DM) were obtained under exogenously applied ABA, while an opposite effect was recorded under exogenously applied fluridone (Flu). The positive impacts of ABA were more predominant in cv. Jundan 20 (JD20) while more negative effects of Flu in cv. Zhengdan 958 (ZD958) as compared with their untreated counterparts (Table 1). Thus, exogenously applied ABA obviously enhanced the responses to drought especially in a drought sensitive cultivar (JD20), while the abscisic acid synthesis inhibitor Flu diminished the responses to drought, especially in the drought resistant cultivar (ZD958) in terms of plant growth and water relations, but these effects were non-significant under well-watered conditions. Perhaps ABA was involved in the regulation of water relations and plant growth in maize plants under drought [2,8,19].

It is well known that glycinebetaine (GB) accumulates in many drought-stressed plants including maize [7]. Glycinebetaine is synthesized from its precursor choline by a two-step oxidation via betaine aldehyde catalyzed by choline monooxygenase (CMO), a ferredoxindependent soluble Rieske-type protein, betaine aldehyde dehydrogenase (BADH), and soluble NAD^+^ dependent enzyme [9]. Some researchers pointed out that their results were consistent with an adaptive value for betaine accumulation in barley (*Hordeum vulgare* L.) during prolonged water stress and in sugar beet (*Beta vulgaris* L.) under salinity. Considerable genetic variation in betaine-accumulating potential in them was also reported [27,28]. However, there are not sufficient data to prove a clear GB metabolism in drought-stressed maize plants [7]. The current experiments have shown that the IR-DS increased BADH activity and choline content, as well as induced high accumulation of GB, especially in a drought resistant cultivar (ZD958). Such increases in both BADH activity and choline content were correlated with enhanced accumulation of GB in the maize plants in response to DS (Figure 2C,D).

A widely reported adaptation in plants to counteract abiotic stress is high accumulation of stress hormones and compatible organic solutes [24,29,30]. Among these substances, ABA is a commonly occurring plant growth regulator in plants actively involved in the control of plant growth and development under drought conditions. For example, the role of ABA in closing stomata of drought-stressed plants has been widely reported [17,18]. This effect is suggested to be vital for fast growth resumption and recovery of water content of plants [23]. Additionally, GB is a major organic osmolyte that accumulates in a variety of plant species in response to different stresses. The accumulation of GB has been found in many organisms, including higher plants. GB has been widely reported to play a part in the tolerance mechanism against a stress [7]. Since accumulation of GB in plant tissues serves as an index of the internal water status of plants, increased leaf RWC in crop plants under drought stress suggests that GB may play a protective role in preventing cell damage from stress-induced dehydration [7,9]. However, a relationship between ABA and drought stress in promoting GB accumulation in maize is not well understood [24]. In the current study, the contents of both ABA and GB were found to be increased in drought-stressed maize seedlings, but the peak of ABA accumulation occurred earlier than GB under the drought regime. These responses in a drought-resistant cultivar, ZD958, were stronger than those in the drought-sensitive JD20 (Figure 1).

Additionally, exogenous ABA imposed greater positive impacts of ABA and GB accumulation in the drought-sensitive cultivar, and the abscisic acid synthesis inhibitor Flu had negative effects on the drought resistant cultivar (Figure 2A,B).

Plant growth inhibition and relative adaptive physiological processes such as osmotic regulation caused by environmental stress might be regulated by the application of plant hormones [7,19,20,24,29,30]. There are many reports demonstrating positive effects of exogenous ABA and the ABA synthesis inhibitor Flu on water relations, plant growth and final crop yield under water deficit conditions [13,16,] (Table 1). The ABA treatments increased GB accumulation and the BADH amount, activity as well as its expression, e.g., in barley under osmotic stress [31], in pear (*Pyrus pyrifolia*) under drought stress [32] and sorghum (*Sorghum bicolor*) under salinity treatment [33]. However, some previous reports showed that ABA may not be associated with osmotic stress-induced betaine accumulation in oat (*Avena sterilis*) and periwinkle (*Vinca major*) [34,35]. The different response to ABA treatment with different plant species may be dependent on their cell signaling pathway in response to environmental stress. Thus, the drought-induced betaine accumulation may involve probably either in ABA-dependent or -independent pathways, which may differ among plant species [36]. Up to now, differential effects of ABA and Flu on modulation of GB metabolism in relation to BADH activity and choline content in maize are still not clear [24]. In our experiments, it has been found that exogenously applied ABA and Flu increased and decreased BADH activity (no effect on choline content), respectively, thereby regulating GB accumulation under DS. The leaf water relations and shoot DM production were correspondingly improved in the maize plants. The greater positive/less negative responses due to exogenous ABA/Flu occurred in JD20 as compared with ZD958. It is, therefore, concluded that endogenous ABA was probably involved in the regulation of GB metabolism by increasing BADH activity only, as well as improving water relations and plant growth under drought, especially in the drought sensitive cultivar (Jundan 20). Moreover, significant correlations among GB metabolism parameters, DM production and leaf RWC were evident in maize plants under DS, but no or less significant under control treatments (Table 2). These results show that contents of ABA, GB and choline as well as BADH activity could be used as potential selection criteria for drought tolerance in maize. Additionally, the effects of exogenously applied ABA and Flu on the above mentioned parameters were highly significant. It is, therefore, suggested that optimal application of plant hormone dose can benefit plant growth under water deficit conditions, but this response is cultivar-specific as is evident from data reported in Table 3.

## 3. Materials and Methods

### 3.1. Plant Material and Trial Location

Hydroponic experiments were performed in a controlled growth chamber at the College of Life Sciences of Northwest A & F University, Yangling, China. The seeds of two maize (*Zea mays* L.) cultivars Zhengdan 958 and Jundan 20 were supplied for the present experiments by the Agronomy College of the same University. According to the previous hydroponic experiment and a field experiment carried out in the farm belonging to the same university, dry matter production and grain yield of the Zhengdan 958 cultivar were relatively little affected by drought [25,26].

### 3.2. Plant Growth and Experimental Design

The seeds of both maize cultivars were germinated at 28 °C for 72 h in the dark. The young seedlings were inserted into holes of styrofoam boards placed in plastic containers (inner length: 26 cm; width 18 cm; height 12 cm) containing treatment solutions. The experimental units were placed in the growth chamber under the following environmental conditions: average day/night temperature 25/18 °C, relative humidity 60–70%, light intensity 350 μmol/m^2^/s and 16 h of photoperiod. The containers were wrapped with black plastic to protect roots from light. Four and eight days after placement of the seedlings in deionized water, the deionized water was replaced by one-half-strength and complete nutrient solution [37], respectively, which contained all essential mineral nutrients for plant growth. The pH of the nutrient solution was adjusted to 6.30 (±0.05) every day.

When seedlings were at the stage of three-leaf, drought stress (DS) treatment started by adding 120 g/kg (w/w) polyethylene glycol (PEG-6000) to achieve drought (osmotic) stress level of approximately −0.23 MPa, dissolved in full strength nutrient solution [38]. The nutrient solution without PEG-6000 served as the control (CK). For each water stress treatment, the seedlings were fed with nutrient solution containing 100 μmol/L ABA or 10 μmol/L fluridone (Flu). The experimental design consisted of six treatments: (1) Control; (2) 12% (w/v) PEG-6000 (PEG); (3) control plus 100 μmol/L ABA (Control + ABA); (4) 12% (w/v) PEG-6000 plus 100 μmol/L ABA (PEG + ABA); (5) control plus 10 μmol/L fluridone (Control + Flu); (6) 12% (w/v) PEG-6000 plus 10 μmol/L fluridone (PEG + Flu).

To explore the relationship between ABA and drought stress (DS) in promoting glycinebetaine (GB) accumulation in maize plants, a second experiment was carried out using both maize cultivars Zhengdan 958 and Jundan 20 under the two treatments, control and 12% (w/v) PEG-6000 in nutrient solution.

Plants were grown in a growth chamber in 3.4 L plastic pots, which were sealed carefully to avoid evaporation, with a sponge wrapped around the interface of the roots and the shoots. All treatment units were replicated four times, with a completely randomized design. The treatment solutions were aerated twelve hours a day.

All experiments were repeated twice. Data presented here are means of four replicates of the two experiments (*n* = 8).

### 3.3. Sampling and Recording of Data

The maize plants were harvested after 12 days of the onset of drought or ABA or Flu treatments in the first experiment and after 0, 12, 24, 36, 48, 60 and 72 h after the onset of drought in the second experiment, respectively. Fresh mass of the shoots was recorded. Then all fresh samples were placed in an oven at 105 °C for 30 min, and then dried to a constant weight at 75 °C.

Relative water content (RWC) was determined using fresh weight (FW), dry weight (DW) and the turgid weight (TW). RWC was calculated from the equation described by Gao [39]: RWC (%) = (FW − DW)/(TW − DW)× 100.

Glycinebetaine (GB) content was determined following Grieve and Grattan [40] with some modifications. Dried and finely powdered plant materials (0.5 g) were shaken with 20 mL of deionized water for 24 h at 25 °C. The extracts were diluted 1:1 with 2 N H_2_SO_4_. Aliquots of 0.5 mL were put in test tubes and cooled in ice water for 1 h, before a cold KI-I_2_ reagent (200 μL) was added. The tubes were stored at 0–4 °C for 16 h and then centrifuged at 12,000× g for 15 min at 4 °C. The supernatant was aspirated. The periodite crystals were dissolved in 5 mL of 1, 2-dichloroethane. After 2–2.5 h, the absorbance was read at 365 nm with a UV–visible spectrophotometer. Reference standards of GB (50–200 g/mL) were used for calibration and estimation of GB concentration in unknown samples. The GB content was expressed as nmol/g DW.

BADH activity was assayed as described by Daniell *et al.* [41] with some modifications. To obtain crude protein extracts, plant materials were homogenized in 250 μL homogenization buffer containing 50 mM HEPES-KOH, pH 8.0, 1 mM EDTA, 20 mM sodium metabisulfite, 10 mM sodium borate, 5 mM ascorbic acid, 5 mM dithiothreitol, and 2% (w/v) PVPP. The homogenates were then centrifuged at 12,000× g for 15 min at 4 °C and the supernatants used for determination of BADH activity. The BADH activity was assayed by monitoring the absorbance at 340 nm with 0.05 mM betaine aldehyde chloride as a substrate. The activity was calculated using the extinction coefficient of 6220/M/cm for NADH. The BADH activity was expressed as μmol/min/mg protein. Protein concentration of the crude extract was measured by the method of Gao [39] using bovine serum albumin as a standard.

Choline content was determined following Feng and Ren [42] and Richard and Emily [43] with some modifications. Dried and finely powdered plant materials (0.5 g) were added with 70 mL of deionized water in a triangle vase (100 mL volume) and shaken up. The vase was bathed in hot water at 80 °C for 15 min, shaken properly with an oscillator for 30 min, set to 100 mL volume scale, and then filtered through a filter paper. Aliquots of 10 mL filtered solution were poured into a triangle vase, cooled in ice water to −5 °C before adding 15 mL Reinecke salt- methanol solution (4 g Reinecke salt was dissolved in 100 mL methanol). The mixed solution was stirred for 30 min and placed in a refrigerator for 12 h. The red water insoluble substance was filtered out, washed with 10 mL propylalcohol three times, dissolved with acetone, and set to 25 mL volume scale. The choline content was assayed by monitoring the absorbance at 520 nm with UV-visible spectrophotometer using acetone as a control. Reference standards of choline (9–27 mg/mL) were used for calibration and estimation of choline concentration in the unknown samples. The choline content was expressed as nmol/g DW.

The content of ABA was assayed in plant leaves by ELISA (enzyme-linked immunosorbent assay) using a monoclonal antibody (MAB) raised against ABA. The polyclonal antibody (RAMIG) was raised against the mouse immunoglobulins and ABA labeled with alkaline phosphatase, according to Weiler [44]. The ABA content was expressed as nmol/g DW.

### 3.4. Statistical Analysis

The data for all attributes were analyzed statistically to work out analysis of variance using the SAS software package [45]. The analysis of variance (ANOVA) was followed by the least significance test (LSD) to determine the significant difference among the mean values at the 0.05 level.

## 4. Conclusions

The results presented in this paper provide evidence that both BADH activity and choline content were involved for enhanced accumulation of GB in maize plants under drought stress. The endogenous ABA seemed to be involved in modulating GB accumulation by enhancing BADH activity, thereby improving leaf RWC and enhancing shoot DM in drought-stressed maize plants, especially in the drought sensitive cultivar (JD20). The peak of ABA content reached earlier than that of GB in the leaves of drought-stressed maize plants. Such, endogenous ABA probably played a positive role as a signal in the regulation of GB metabolism, water relations and plant growth by regulating BADH activity, but not choline content. The above results have proved that the drought-induced GB accumulation in maize may be involved probably in ABA-dependent pathway. The exogenous ABA provided some protection against the DS effects on maize plants by regulating endogenous ABA level and GB metabolism.

## Figures and Tables

**Table 1:** Modulation effect of abscisic acid (ABA) on dry matter (DM) and leaf relative water content (RWC) of maize seedlings under drought stress.

Treatment	DM (g/plant)		RWC (%)	
	
	Zhengdan 958	Jundan 20	Zhengdan 958	Jundan 20
Control	0.362 ± 0.011 ^a^	0.397 ± 0.013 ^a^	91.78 ± 1.20 ^a^	92.11 ± 1.12 ^a^
	(B)	(A)	(A)	(A)
PEG	0.268 ± 0.012 ^b^	0.239 ± 0.015 ^b^	82.83 ± 1.15 ^b^	79.01 ± 1.15 ^b^
	(A)	(B)	(A)	(B)
Control + ABA	0.347 ± 0.021 ^a^	0.394 ± 0.010 ^a^	90.17 ± 1.46 ^a^	91.09 ± 1.25 ^a^
	(B)	(A)	(A)	(A)
PEG + ABA	0.311 ± 0.007 ^c^	0.288 ± 0.009 ^c^	86.77 ± 1.50 ^c^	83.02 ± 1.07 ^c^
	(A)	(B)	(A)	(B)
Control + Flu	0.338 ± 0.011 ^ab^	0.381 ± 0.014 ^a^	91.12 ± 1.65 ^a^	92.32 ± 1.55 ^a^
	(A)	(A)	(A)	(A)
PEG + Flu	0.225 ± 0.008 ^d^	0.204 ± 0.011 ^d^	79.22 ± 1.75 ^d^	76.02 ± 1.60 ^d^
	(A)	(B)	(A)	(B)

Mean values in the same column followed by the same letters within variables are not significantly different among six treatments, and the same row followed by the same letters in parentheses between the two cultivars with the same water and ABA or Flu treatment. Letters (a–c) showing least significant difference between treatments and cultivars at the 0.05 level.

**Table 2:** Correlation coefficients of dry matter (DM), leaf relative water content (RWC), ABA content (ABAC), GB content (GBC), choline content (CC) and BADH activity (BADHA) of both cultivars under drought stress (above diagonal) and control condition (below diagonal).

Parameter	DM	RWC	ABAC	GBC	CC	BADHA
DM		0.946 ***	0.930 ***	0.941 ***	0.684 **	0.943 ***
RWC	0.356		0.912 ***	0.988 ***	0.789 ***	0.974 ***
ABAC	0.422	0.122		0.941 ***	0.501 *	0.888 ***
GBC	0.567 *	0.516 *	0.375		0.915 ***	0.978 ***
CC	0.448	0.422	0.374	0.739 ***		0.808 ***
BADHA	0.208	0.342	0.251	0.705 **	0.611 **	

*, **, *** significant at *P* < 0.05, 0.01, 0.001, respectively.

**Table 3:** *F* values of the effects of exogenous ABA (A) or fluridone (Flu) treatment, cultivar (Cv), water regime (W) and their interactions on dry matter (DM), leaf relative water content (RWC), ABA content (ABAC), GB content (GBC), choline content (CC) and BADH activity (BADHA).

Variation	W	Cv	A	W × Cv	W × A	Cv × A	W × Cv × A
DM	1133.5 ***	5.8 *	42.6 ***	128.5 ***	86.6 ***	8.9 *	8.5 *
RWC	3129.9 ***	496.2 ***	532.6 ***	205.5 ***	619.7 ***	9.3 **	9.7 **
GBC	3406.9 ***	261.2 ***	276.8 ***	217.7 ***	290.6 ***	11.7 **	12.9 **
ABAC	15932.8 ***	140.7 ***	829.1 ***	83.6 ***	220.5 ***	10.07 **	10.07 **
CC	3982.9 ***	258.3 ***	10.9 **	215.1 ***	34.4 ***	0.03	0.01
BADHA	2344.0 ***	48.5 ***	53.1 ***	17.5 ***	67.4 ***	3.4	0.34
	**W**	**Cv**	**Flu**	**W × Cv**	**W × Flu**	**Cv × Flu**	**W × Cv × Flu**
DM	613.7 ***	5.83 *	97.4 ***	9.3 **	12.2 **	8.0 *	12.0 **
RWC	6798.1 ***	308.4 ***	381.6 ***	159.7 ***	74.3 ***	56.5 ***	10.6 **
GBC	5641.3 ***	264.6 ***	283.0 ***	246.4 ***	212.8 ***	8.3 *	9.9 **
ABAC	12090.5 ***	86.4 ***	1390.9 ***	80.2 ***	868.6 ***	27.2 ***	8.0 *
CC	1751.4 ***	70.7 ***	4.17 *	80.2 ***	13.7 **	10.5 **	2.6
BADHA	2611.6 ***	137.0 ***	244.2 ***	5.6 *	5.1 *	35.6 **	4.1

*, **, *** significance at *P* < 0.05, 0.01, 0.001, respectively.

## References

[1] Li S.X. (2007). Dry Land Agriculture in China.

[2] Zhang L.X., Li S.X., Zhang H., Liang Z.S. (2007). Nitrogen rates and water stress effects on production, lipid peroxidation and antioxidative enzyme activities in two maize (*Zea mays* L.) genotypes. J. Agron. Crop Sci.

[3] Lu G.H., Ren D.L., Wang X.Q., Wu J.K., Zhao M.S. (2010). Evaluation on drought tolerance of maize hybrids in China. J. Maize Sci.

[4] Ashraf M. (2010). Inducing drought tolerance in plants: Some recent advances. Biotechnol. Adv.

[5] Mittler R. (2002). Oxidative stress, antioxidants and stress tolerance. Trends Plant Sci.

[6] Zhang L.X., Li S.X., Liang Z.S., Li S.Q. (2009). Effect of foliar nitrogen application on nitrogen metabolism, water status and plant growth in two maize (*Zea mays* L.) cultivars under short-term moderate stress. J. Plant Nutr.

[7] Ashraf M., Foolad M.R. (2007). Roles of glycinebetaine and proline in improving plant abiotic stress resistance. Environ. Exp. Bot.

[8] Zhang L.X., Li S.X., Liang Z.S. (2009). Differential plant growth and osmotic effects of two maize (*Zea mays* L.) cultivars to exogenous glycinebetaine application under drought stress. Plant Growth Regul.

[9] Rhodes D., Hanson A.D. (1993). Quaternary ammonium and tertiary sulfonium compounds in higher-plants. Annu. Rev. Plant Physiol. Plant Mol. Biol.

[10] Sakamoto A., Murata N. (2002). The role of glycine betaine in the protection of plants from stress: Clues from transgenic plants. Plant Cell Environ.

[11] Sithtisarn S., Harinasut P., Pornbunlualap S., Cha-Um S. (2009). Accumulation of glycinebetaine and betaine aldehyde dehydrogenase activity in *Eucalyptus camaldulensis* clone T5 under *in vitro* salt stress. Kasetsart J. (Nat. Sci.).

[12] Jakab G., Ton J., Flors V., Zimmerli L., Métraux J., Mauch-Mani B. (2005). Enhancing Arabidopsis salt and drought stress tolerance by chemical priming for its abscisic acid responses. Plant Physiol.

[13] Jiang W., Lafitte R. (2007). Ascertain the effect of PEG and exogenous ABA on rice growth at germination stage and their contribution to selecting drought tolerant genotypes. Asian J. Plant Sci.

[14] Maleki M., Ebrahimzade H., Gholami M., Niknam V. (2011). The effect of drought stress and exogenous abscisic acid on growth, protein content and antioxidative enzyme activity in saffron (*Crocus sativus* L.). Afr. J. Biotechnol.

[15] Kurahashi Y., Terashima A., Takumi S. (2009). Variation in dehydration tolerance, ABA sensitivity and related gene expression patterns in D-Genome progenitor and synthetic hexaploid wheat lines. Int. J. Mol. Sci.

[16] Hooker S.T., Thorpe A.T. (1998). Effects of fluridone and abscisic acid on lateral root initiation and root elongation of excised tomato roots cultured *in vitro*. Plant Cell Tissue Org.

[17] Ruggiero B., Koiwa H., Manabe Y., Quist T.M., Inan G., Saccardo F., Joly R.J., Hasegawa P.M., Bressan R.A., Maggio A. (2004). Uncoupling the effects of abscisic acid on plant growth and water relations. Analysis of *sto1/nced3*, an abscisic acid-deficient but salt stress tolerant mutant in Arabidopsis. Plant Physiol.

[18] Jiang F., Hartung W. (2007). Long-distance signalling of abscisic acid (ABA): The factors regulating the intensity of the ABA signal. J. Exp. Bot.

[19] Achard P., Cheng H., de Grauwe L., Decat J., Schoutteten H., Moritzn T., van Der Straeten D., Peng J.R., Harberd N.P. (2006). Integration of plant responses to environmentally activated phytohormonal signals. Science.

[20] Maggio A., Barbieri G., Raimondi G., Pascale S.D. (2010). Contrasting effects of GA_3_ treatments on tomato plants exposed to increasing salinity. J. Plant Growth Regul.

[21] Zeevart J.A.D., Creelman R.A. (1998). Metabolism and physiology of abscisic acid. Annu. Rev. Plant Physiol.

[22] Ren H., Zhihui G., Lin C., Kaifa W., Jing L., Yijuan F., William J.D., Wensuo J., Jianhua Z. (2007). Dynamic analysis of ABA accumulation in relation to the rate of ABA catabolism in maize tissues under water deficit. J. Exp. Bot.

[23] Zhang J.H., Jia W.S., Yang J.C., Ismail A.M. (2006). Role of ABA in integrating plant responses to drought and salt stresses. Field Crops Res.

[24] Hancock J.T., Neill S.J., Wilson I.D. (2011). Nitric oxide and ABA in the control of plant function. Plant Sci.

[25] Zhang L.X., Gao M., Li S.Q., Li S.X., Liang Z.S. (2011). Water status and photosynthesis in two maize (*Zea Mays* L.) cultivars as affected by supplied nitrogen form and drought stress. Pak. J. Bot.

[26] Zhang Y.K., Wang L.X., Yang J.H., Liang D.J., Wang X.L., Xi L.Y. (2007). China maize potential yield developing technique advanced. Chin. Agri. Sci. Bull.

[27] Hattori T., Mitsuya S., Fujiwara T., Jagendorf A.T., Takabe T. (2009). Tissue specificity of glycinebetaine synthesis in barley. Plant Sci.

[28] Hanson A.D., Wyse R. (1982). Biosynthesis, translocation, and accumulation of betaine in sugar beet and its progenitors in relation to salinity. Plant Physiol.

[29] Ashraf M., Harris P.J.C. (2004). Potential biochemical indicators of salinity tolerance in plants. Plant Sci.

[30] Taiz L., Zeiger E (2002). Plant Physiology.

[31] Ishitani M., Nakamura T., Han S.Y., Takabe T. (1995). Expression of the betaine aldehyde dehydrogenase gene in barley in response to osmotic stress and abscisic acid. Plant Mol. Biol.

[32] Gao X.P., Pan Q.H., Li M.J., Zhang L.Y., Wang X.F., Shen Y.Y., Lu Y.F., Chen S.W., Liang Z., Zhang D.P. (2004). Abscisic acid is involved in the water stress-induced betaine accumulation in pear leaves. Plant Cell Physiol.

[33] Saneoka H., Ishiguro S., Moghaieb R.E.A. (2001). Effect of salinity and abscisic acid on accumulation of glycinebetaine and betaine aldehyde dehydrogenase mRNA in Sorghum leaves (*Sorghum bicolor*). J. Plant Physiol.

[34] Maldonado C.A., Zuniga G.E., Corcuera L.J., Alberdi M. (1997). Effect of water stress on frost resistance of oat leaves. Environ. Exp. Bot.

[35] Rai S.P., Luthra R., Gupta M.M., Kumar S. (2001). Pleiotropic morphological and abiotic stress resistance phenotypes of the hyper-abscisic acid producing *Abo*-mutant in the periwinkle *Catharanthus roseus*. J. Biosci.

[36] Shinozaki K., Yamaguchi-Shinozaki K. (1997). Gene expression and signal transduction in water-stress response. Plant Physiol.

[37] Hoagland D.R., Arnon D.I. (1950). The water culture method for growing plants without soils. Calif. Agric. Exp. Sta. Cir.

[38] Wang J., Li D.Q. (2002). Effects of water stress on AsA-GSH cycle and H_2_O_2_ content in maize root. Chin. J. Eco-Agric.

[39] Gao J.F. (2000). Experiment Technique of Plant Physiology.

[40] Grieve C.M., Grattan S.R. (1983). Rapid assay for determination of water soluble quaternary ammonium compounds. Plant Soil.

[41] Daniell H., Muthukumar B., Lee S.B. (2001). Marker free transgenic plants: Engineering the chloroplast genome without the use of antibiotic selection. Curr. Genet.

[42] Feng Z.X., Ren A.N. (2004). Mensuration of the content of choline in diospyros leaves. Tianjin J. Tradit. Chin. Med.

[43] Richard J.W., Emily R.M. (1945). Spectrophotometric determination of small amounts of choline. J. Biol. Chem.

[44] Weiler E. (1982). An enzyme-immunoassay for *cis*-(+)-abscisic acid. Physiol. Plant.

[45] (1996). *SAS Software*, version 8.2.

